# In Situ Raman Spectroscopy-Enabled Microfluidic Gel Chromatography for Revealing Real-Time Separation Dynamics of Single-Walled Carbon Nanotubes

**DOI:** 10.3390/polym17010093

**Published:** 2025-01-01

**Authors:** Byeongji Beom, Seung-Chan Jung, Wonjun Jang, Jong-Keon Won, Jihoon Jeong, Yu-Jeong Choi, Man-Ki Moon, Jae-Hee Han

**Affiliations:** Department of Materials Science and Engineering, Gachon University, Seongnam 13120, Republic of Korea; jjkk3239@gachon.ac.kr (B.B.); jsc4486@gachon.ac.kr (S.-C.J.); juni1205@gachon.ac.kr (W.J.); wjk0508@gachon.ac.kr (J.-K.W.); wlgns9751@gachon.ac.kr (J.J.); dbwjd330@gachon.ac.kr (Y.-J.C.); wangpeterpan@gachon.ac.kr (M.-K.M.)

**Keywords:** single-walled carbon nanotube (SWNT) separation, microfluidic gel chromatography, in situ Raman spectroscopy, electronic-type separation, separation dynamics

## Abstract

Single-walled carbon nanotubes (SWNTs) exhibit distinct electronic properties, categorized as metallic or semiconducting, determined by their chirality. The precise and selective separation of these electronic types is pivotal for advancing nanotechnology applications. While conventional gel chromatography has been widely employed for large-scale separations, its limitations in addressing microscale dynamics and electronic-type differentiation have persisted. Here, we present a polydimethylsiloxane (PDMS)-based microfluidic gel chromatography platform, coupled with real-time in situ Raman spectroscopy, designed to achieve the high-resolution electronic-type separation of SWNTs. This platform systematically isolates metallic- and semiconducting-enriched fractions (M1–M3 and S1–S3) while quantitatively analyzing separation dynamics through G-band spectral shifts and G^−^/G^+^ intensity ratios. By normalizing the SDS concentration and calculating rate constants, we reveal the intrinsic elution kinetics of SWNTs, with metallic fractions exhibiting faster elution dynamics compared to their semiconducting counterparts. Our approach bridges the gap between microscale precision and industrial scalability, emphasizing the critical role of dispersant concentration in fine-tuning separation outcomes. This advancement not only resolves the challenges of electronic-type differentiation but also demonstrates the versatility of PDMS microfluidic systems in delivering real-time insights into nanomaterial purification processes. By integrating continuous dynamic analysis with gel chromatography, this study establishes a transformative framework for scaling nanomaterial separations and unlocking new potential in chirality-specific applications.

## 1. Introduction

Carbon nanotubes (CNTs), as one of the most remarkable carbon allotropes, have gained significant attention due to their extraordinary properties and unique cylindrical structure, which is formed by rolling a single graphene sheet into a seamless tubular configuration. Among them, single-walled carbon nanotubes (SWNTs) are particularly notable for their nanoscale diameter, exceptional electrical conductivity, and remarkable mechanical strength [1,2]. These properties are inherently linked to their chirality, defined by the chiral indices (n,m), which determine their electronic nature. SWNTs exhibit metallic behavior when $\left(n-m\right)mod3=0$, while other configurations demonstrate semiconducting characteristics with varying bandgaps, depending on chirality [3,4]. This intrinsic dichotomy in electronic properties makes the selective separation of SWNTs critical for advanced applications, ranging from nanoelectronics to energy storage systems, such as transistors, sensors, and energy storage systems [5,6,7,8,9,10,11]. Despite considerable progress in synthesis techniques, the production of SWNTs typically yields mixtures of metallic and semiconducting nanotubes, which hinders their direct utilization in targeted applications. Consequently, a variety of post-synthesis separation methods—such as gel chromatography, density gradient centrifugation, and electrophoresis—have been developed to address this challenge [12,13].

**Figure 1:**
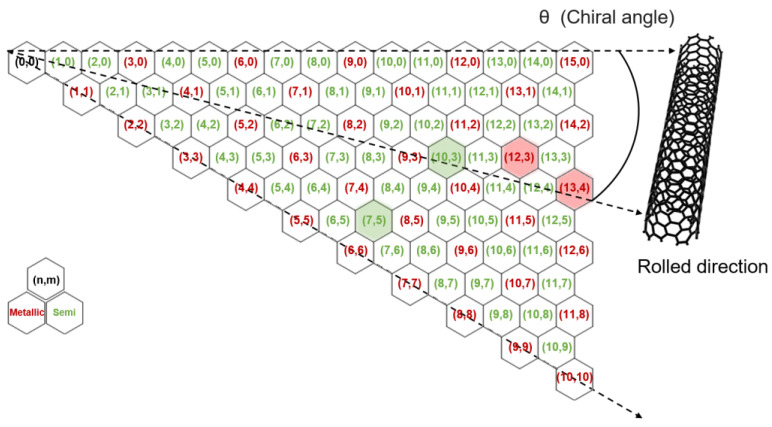
Map illustrating the structural and electronic characteristics of SWNTs based on their chirality. The hexagonal lattice defines the relationship between chiral indices (*n, m*), chiral angle (*θ*), and rolled direction, which collectively determine whether a given SWNT is metallic (red) or semiconducting (green). Four specific chiralities—(13,4), (12,3), (10,3), and (7,5) are highlighted. These chiralities are later used to analyze relative intensity ratios and provide insight into the selective separation of metallic and semiconducting-enriched SWNTs.

Among these, gel chromatography has emerged as a promising technique for large-scale, chirality-specific separation, leveraging the differential affinities of SWNTs to the gel matrix, as shown in Figure 1, depending on their electronic properties. However, while effective for bulk separations, the underlying mechanisms governing SWNT–gel interactions remain poorly understood, limiting its optimization for finer, microscale precision [13,14,15,16,17,18].

Microscale separation offers a novel opportunity to investigate the intricate dynamics of SWNT interactions with unmatched precision, enabling the real-time observation of molecular behaviors and separation processes. This study pioneers a polydimethylsiloxane (PDMS)-based microfluidic gel chromatography platform specifically designed for the high-resolution electronic-type separation of SWNTs. PDMS, known for its ease of fabrication and compatibility with microfluidic designs, provides a robust framework for constructing channels that replicate traditional chromatographic conditions. By integrating in situ Raman spectroscopy, the platform allows the systematic isolation of metallic- and semiconducting-enriched fractions (M1–M3 and S1–S3), capturing dynamic shifts in G-band characteristics and G^−^/G^+^ intensity ratios to reveal the intrinsic elution kinetics of SWNTs [19,20]. The integration of gel chromatography with in situ Raman spectroscopy in this system addresses key limitations of bulk-scale techniques by providing dynamic, high-resolution insights into electronic-type separations at the microscale. Metallic SWNTs exhibit faster elution dynamics compared to semiconducting ones, a distinction further elucidated by normalizing elution kinetics to SDS concentration. This approach highlights the interplay between SDS concentration, flow dynamics, and electronic properties, enabling the systematic fine-tuning of separation outcomes. Such insights are vital for overcoming long-standing challenges in achieving chirality-specific SWNT separations. By bridging the gap between large-scale separation methods and the precision required for nanoscale studies, this study establishes a comprehensive framework for advancing SWNT purification technologies. The platform’s scalability and adaptability not only make it a valuable tool for fundamental research but also position it as a robust solution for industrial applications. By leveraging the unique advantages of microfluidics, this work sets a new benchmark for developing next-generation nanomaterial separation methodologies, paving the way for transformative advancements in the field. In addition, to enhance clarity, all abbreviations utilized throughout this manuscript are comprehensively defined in Table S1, which is included in the Supplementary Materials.

## 2. Materials and Methods

### 2.1. Materials

In this study, purified HiPco SWNTs (Nanointegris, HP34-093, Boisbriand, QC, Canada) were used for separation. Sodium dodecyl sulfate (SDS, ≥98.5%, Sigma-Aldrich, Cat. No. L3771, Saint Louis, MO, USA) was used for dispersing the SWNTs. Disposable 10 mL Polypropylene Columns (Thermo Fisher Scientific, Cat. No. 29924 Waltham, MA, USA) were purchased and used along with polypropylene columns and polyethylene separators for SWNT separation. Sephacryl™ S-200 HR gel (Cytiva, Washington, DC, USA) was used to separate the SWNTs. The PDMS mold was prepared using Sylgard™ 184 (Dow Inc., Midland County, MI, USA) with the provided curing agent.

### 2.2. Dispersion of SWNTs

SWNTs were dispersed in 0.5 wt% SDS at a concentration of 1 mg/mL. Tip sonication was used for 5 h for dispersion. The dispersed SWNTs solution was then centrifuged at 32,000 rpm to remove impurities and metal catalysts remaining after synthesis. Only the top 20% supernatant of the solution was collected and used for separation.

### 2.3. Gel Chromatography

#### 2.3.1. Conventional Column Method

**Figure 2:**
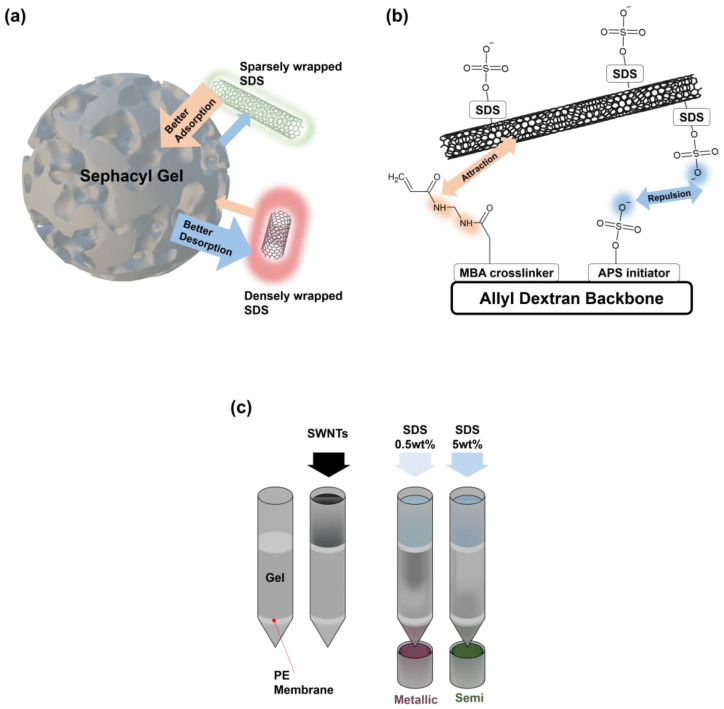
(**a**) Schematic representation of the SDS–SWNT–gel system and the separation mechanism employed in gel chromatography. The extent of SDS wrapping influences the interaction of SWNTs with the Sephacryl gel. Sparsely wrapped SWNTs (green) exhibit enhanced adsorption due to reduced repulsive forces, while densely wrapped SWNTs (red) desorb more readily because of stronger repulsion. (**b**) Molecular structure of the Sephacryl gel, illustrating functional groups derived from the MBA crosslinker and APS initiator attached to an allyl dextran backbone. These functional groups create a balance of attractive and repulsive forces that selectively interact with SDS-wrapped SWNTs. (**c**) Conventional gel chromatography process, demonstrating the sequential separation of metallic and semiconducting SWNTs by modulating SDS concentrations (0.5 wt% for metallic SWNTs and 5 wt% for semiconducting SWNTs). This process enables the precise differentiation of SWNT types based on their electronic properties.

The conventional column method for separating metallic and semiconducting SWNTs relies on differences in SDS wrapping behavior around the nanotubes. Metallic SWNTs, due to their unique electronic properties, are more effectively wrapped with SDS compared to semiconducting SWNTs, as illustrated in Figure 2a. This differential wrapping enhances their interaction with the Sephacryl gel, facilitating selective adsorption and subsequent desorption. Metallic SWNTs adsorbed onto the gel can be eluted with a lower SDS concentration, whereas higher concentrations are required for semiconducting SWNTs, which interact more strongly with the gel. The functional groups in the gel structure, as depicted in Figure 2b, play a pivotal role in this selective separation. The Sephacryl gel used in this study is a polymer gel constructed on an allyl dextran backbone chain. During the synthesis of the gel beads, ammonium persulfate (APS) acts as the initiator, and *N*,*N*′-methylenebisacrylamide (MBA) serves as the crosslinking agent. These components endow the gel with specific functional groups that are critical to its interaction with SWNTs [21]. In the final synthesized gel, the organosulfate groups originating from APS and the amide groups derived from MBA facilitate interactions between the gel matrix and SWNTs. These functional groups play a critical role in enhancing the compatibility and interaction strength between the gel and the nanotubes, as previously reported [10,22].

The organosulfate groups are negatively charged and interact through electrostatic repulsion with the negatively charged sulfate groups of SDS. This repulsive force is particularly significant for SDS-wrapped SWNTs, whose degree of wrapping modulates their adsorption behavior on the gel. In contrast, the amide groups act as electron donors, creating attractive interactions with the SWNT surface [10]. These opposing forces create a selective adsorption mechanism based on the electronic properties of the SWNTs. Metallic SWNTs, which are typically more sparsely wrapped with SDS, exhibit weaker repulsion from the organosulfate groups, resulting in stronger interactions with the gel matrix. Conversely, semiconducting SWNTs, which are more densely wrapped with SDS, experience greater repulsion, reducing their affinity for the gel. Simultaneously, the degree to which SDS wraps the SWNTs directly influences their desorption behavior. As SDS concentration increases, the interaction between SDS-wrapped SWNTs and the gel is systematically modulated. This mechanism enables the sequential elution of metallic and semiconducting SWNTs through gradual changes in SDS concentration, as illustrated in Figure 2c. The selective interaction between the gel and SWNTs, driven by the balance of repulsive and attractive forces, highlights the efficacy of this system in separating SWNTs based on their electronic properties. [23,24].

Figure 2c outlines the experimental setup for gel chromatography. Ethanol-washed columns with membranes at the base are used to prevent gel leakage. A volume of 4 mL of Sephacryl gel is loaded into the column, followed by the injection of 1 mL of an unseparated SWNT solution. The SWNTs adsorbed onto the gel are eluted sequentially. Metallic SWNTs are first eluted using 1 mL of 0.5 wt% SDS, while semiconducting SWNTs are eluted with 1 mL of 5 wt% SDS following a cleaning step using 5 mL of 0.5 wt% SDS. This stepwise elution process ensures a high degree of separation, with the SDS concentration precisely tuned to exploit the differences in SWNT–gel interactions.

#### 2.3.2. Microchannel Column Method

**Figure 3:**
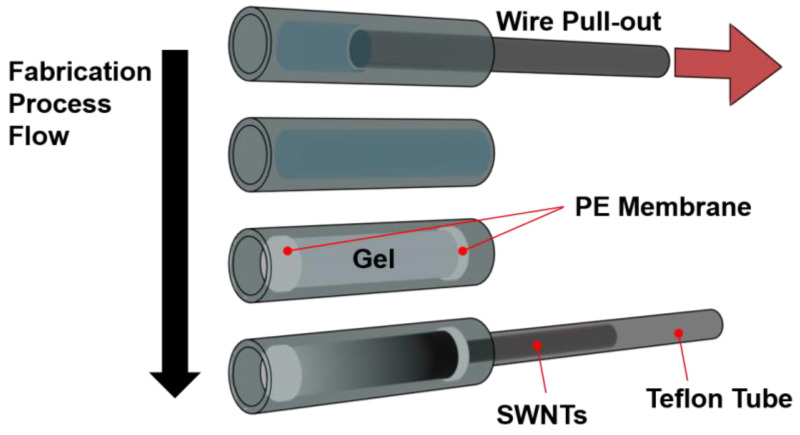
Schematic representation of the fabrication and assembly process for the microfluidic gel chromatography column system using a PDMS mold. The process involves molding the microchannel with a wire template, integrating a polyethylene (PE) membrane to retain the gel, and connecting Teflon tubes for fluid injection. The assembled microchannel enables precise control for SWNT separation via SDS-based gel chromatography.

To fabricate the microchannel system, polydimethylsiloxane (PDMS) was mixed with a curing agent and poured into a mold containing a wire template with a radius of 800 μm. The mixture was degassed under vacuum and subsequently cured at 90 °C for 1 h to achieve crosslinking. Once the curing was complete, the wire was carefully removed, leaving behind a cylindrical microchannel with a radius of 800 μm. As illustrated in Figure 3, the microchannel mold was thoroughly washed with ethanol to remove any residual contaminants. The PE membrane was then placed within the channel, replicating the gel retention setup used in conventional column chromatography. The Sephacryl gel was loaded into the channel, and Teflon tubes were attached to the inlet and outlet of the microchannel to connect it to an external syringe pump. To test the adsorption capability of the gel within the microchannel, a dispersed SWNT solution was injected. Using a syringe pump (Longer, Hebei, China) to simulate the gravitational force in traditional chromatography, SDS solutions of 0.5 wt% and 5 wt% were sequentially introduced to achieve selective elution of metallic and semiconducting SWNTs, respectively. This setup provided a refined and precise means of SWNT separation within a controlled microfluidic environment [25,26,27].

## 3. Results and Discussion

**Figure 4:**
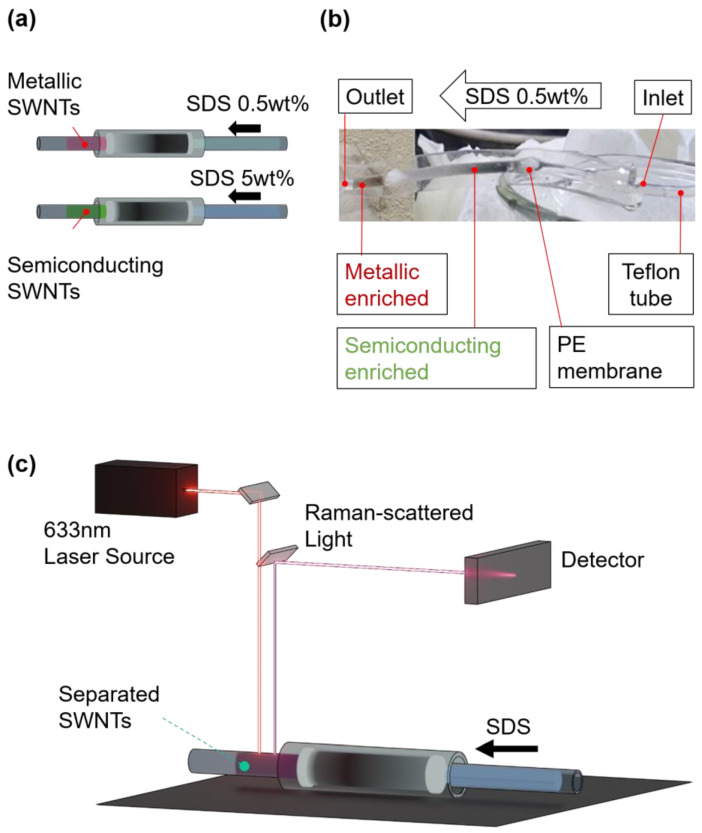
(**a**) Schematic of the microfluidic gel chromatography process used for the separation of metallic and semiconducting SWNTs within a PDMS-based column. The separation was achieved by sequentially injecting SDS dispersant solutions at 0.5 wt% and 5 wt%, facilitating the elution of metallic and semiconducting SWNTs, respectively. (**b**) Photographic evidence of the separated SWNT fractions at the outlet, where metallic SWNTs exhibited a reddish color and semiconducting SWNTs appeared greenish, demonstrating the effectiveness of the separation process. (**c**) Experimental setup for in situ Raman spectroscopy analysis conducted downstream of the microfluidic column, illustrating the in situ characterization of SWNTs via a 633 nm laser source and detector system. The Raman setup enables precise analysis of electronic types based on radial breathing mode (RBM) signals without disrupting the chromatographic environment.

The efficient separation of metallic and semiconducting SWNTs was achieved using a PDMS-based microfluidic gel chromatography column, as depicted in Figure 4a. The process involved injecting sodium dodecyl sulfate (SDS) at two distinct concentrations: 0.5 wt% SDS for metallic SWNTs and 5 wt% SDS for semiconducting SWNTs. This approach capitalized on the differential interactions between SWNTs and the gel matrix, driven by the variation in repulsion forces mediated via the SDS concentration. The stepwise elution enabled clear differentiation between metallic and semiconducting SWNT fractions. The visual contrast between the separated fractions was striking, as shown in Figure 4b. Metallic SWNTs, characterized by their weaker interactions with SDS, exhibited a reddish appearance, whereas semiconducting SWNTs, with stronger gel affinities, displayed a greenish color. This color distinction underscores the precision of the separation method and aligns with the inherent optical properties of HiPco SWNTs. The reddish tint of metallic SWNTs and the greenish tint of semiconducting SWNTs were consistent with prior observations, reaffirming the effectiveness of SDS-mediated chromatographic separation [28].

### 3.1. RBM Analysis

Post-separation analysis of the SWNT fractions was conducted using in situ Raman spectroscopy, as shown in Figure 4c, to precisely determine their electronic structure. The method leveraged the radial breathing mode (RBM) vibrations in the range of 100–300 cm^−1^, which are highly sensitive to the diameter and electronic properties of SWNTs. RBM peaks below 250 cm^−1^ were attributed to metallic SWNTs, while those above this threshold corresponded to semiconducting SWNTs, providing a clear distinction between the two types. To avoid laser-induced disruptions such as heating or photon-induced energy effects, the Raman measurements were performed on SWNT fractions flowing through a Teflon tube connected to the microfluidic column. This approach reinforced the reliability of the microfluidic gel chromatography system in achieving efficient separation and accurate electronic characterization, offering significant insights into SWNT properties for advanced nanomaterial analysis.

**Figure 5:**
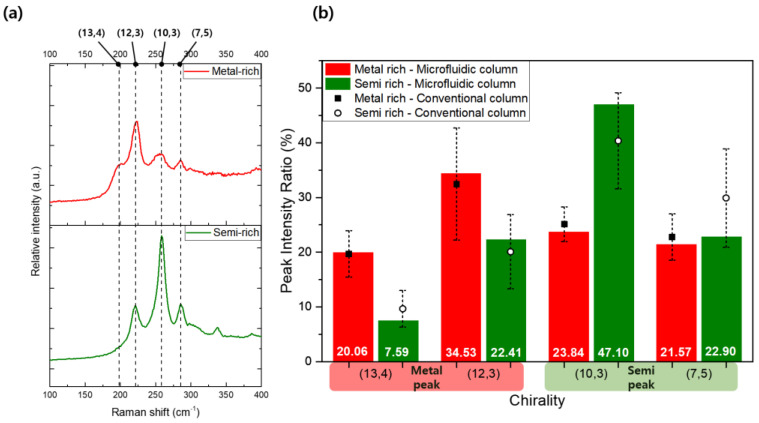
(**a**) Raman spectra in the radial breathing mode (RBM) region for metallic-rich (top) and semiconducting-rich (bottom) SWNT samples, highlighting four distinct chirality peaks: (13,4), (12,3), (10,3), and (7,5). The vertical dashed lines indicate the Raman shifts corresponding to these chiralities. (**b**) Relative peak intensity ratios (%) for the four chiralities of metallic-rich and semiconducting-rich fractions, compared between conventional and microfluidic columns. The differences in intensity ratios demonstrate the effectiveness of the separation process in enriching SWNTs based on their electronic type.

Figure 5a presents the Raman spectra of the metallic-enriched (reddish) and semiconducting-enriched (greenish) samples, highlighting significant differences in the four primary peaks: 197 cm^−1^ for metallic (13,4), 217 cm^−1^ for metallic (12,3), 255 cm^−1^ for semiconducting (10,3), and 284 cm^−1^ for semiconducting (7,5). The distinct RBM peaks observed for these chiralities confirm the successful separation, as the metallic and semiconducting SWNTs exhibit characteristic vibrational modes corresponding to their respective structural configurations. The intensity and position of these peaks provide insight into the composition and purity of the separated samples, with metallic SWNTs showing more prominent low-frequency RBM peaks compared to their semiconducting counterparts. In the metal-rich sample, the (12,3) metallic peak was particularly prominent, indicating a higher concentration of metallic SWNTs with this specific chirality. In contrast, the semi-rich sample exhibited distinct peaks for (10,3) and (7,5) semiconducting SWNTs, with only minor contributions from the (12,3) metallic SWNTs. This suggests that the separation process was effective in enriching the sample with semiconducting SWNTs while significantly reducing the presence of metallic SWNTs, thereby demonstrating the efficacy of the PDMS microchannel-based gel chromatography in achieving selective separation based on electronic properties.

Figure 5b provides a comprehensive comparison of the relative ratios of the four major Raman peaks between the metallic-enriched and semiconducting-enriched SWNT samples. Peaks associated with metallic chiralities, specifically (13,4) and (12,3) SWNTs, were more prominent in the metallic-rich samples. Conversely, peaks linked to semiconducting chiralities, such as (10,3) and (7,5) SWNTs, exhibited higher relative intensities in the semiconducting-enriched samples. Notably, while the (7,5) peak in semiconducting-enriched samples was only marginally more intense than in metallic-enriched samples, the (10,3) peak showed a substantial increase in intensity, underscoring the selective enrichment of semiconducting SWNTs. Compared to relative ratios from the conventional column, overall profiles were consistent with differences between electronic types. Through the analysis of the RBM region, these measurements confirm that the separation process in the microfluidic column effectively altered the relative proportions of metallic and semiconducting SWNTs across the different sample fractions. The observed shift in peak ratios across separated samples highlights the capacity of this micro-sized PDMS column-based gel chromatography system to achieve selective isolation based on SWNT chirality and electronic properties. Additional information on gel chromatography using conventional columns can be found in Figure S1.

### 3.2. G-Band Analysis

**Figure 6:**
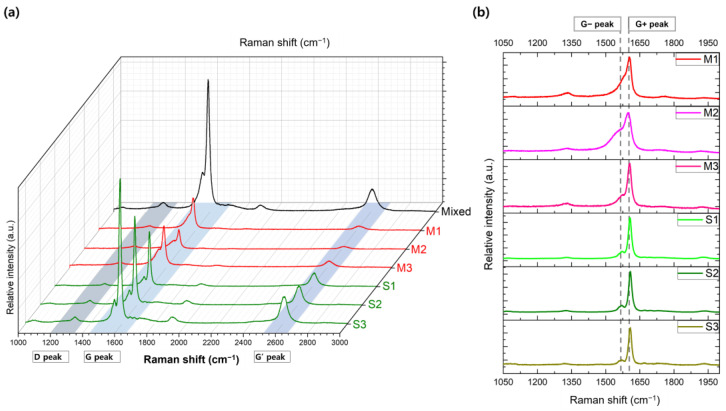
(**a**) Raman spectra comparing metallic-rich (M1–M3) and semiconducting-rich (S1–S3) SWNT fractions across a broad spectral range, including the D, G, and G’ peaks. The stacked plot highlights distinct spectral features corresponding to each separated fraction. (**b**) Zoomed-in view of the G peaks, emphasizing the G^−^ and G^+^ components for each sample. The differences in peak positions and shapes between metallic-rich and semiconducting-rich fractions illustrate the separation’s effectiveness and the electronic-type-specific interactions during the elution process.

Furthermore, the separated SWNT samples were examined for variations in their G peak profiles, as illustrated in Figure 6. The G peak, a hallmark of sp^2^-hybridized carbon structures, provides valuable information on the electronic characteristics of SWNTs. It is typically split into two components: the G^−^ peak, observed around 1560 cm^−1^, and the G^+^ peak, located near 1590 cm^−1^ [29]. The G^−^ peak is associated with atomic vibrations along the nanotube axis, whereas the G^+^ peak corresponds to vibrations in the circumferential direction. In metallic SWNTs, the G^−^ peak is typically broader due to the influence of free electrons, while in semiconducting SWNTs, it appears sharper and more defined, reflecting the absence of free carriers [30].

Figure 6a provides an overview of the Raman spectra, showing the D, G, and G’ peaks across all separated samples. This spectral profile displays the progressive evolution from mixed SWNTs to metal-enriched (M1–M3) and semi-enriched (S1–S3) fractions, facilitating a comparative analysis of the relative intensities at various Raman shifts. This visual comparison highlights the distinct separation of metallic and semiconducting SWNT components achieved through the process. From M1 to S3, increments in the overall intensity of the D and G peaks are observed, which can be attributed to semiconducting SWNTs comprising approximately 73% [31]. As the separation progresses to the later stages, the increased concentration leads to higher intensity. Specifically, Figure 6b offers a close-up of the G peaks, where the G^−^ and G^+^ components show noticeable shifts and shape changes across the samples, further evidencing the separation. The elution fractions collected at different stages were labeled as M1, M2, and M3 (for metallic SWNTs) and S1, S2, and S3 (for semiconducting SWNTs). In Figure 6b, a clear trend is visible in the G^−^ peak profiles, evolving from a broad shape in M1 to a sharp form in S3, which indicates a continuous enrichment of semiconducting SWNTs through the separation process. The broader G^−^ peaks observed in the M1, M2, and M3 samples underscore their metallic nature, while the progressively narrow G^−^ peaks in S1, S2, and S3 confirm the increasing purity of semiconducting SWNTs in these later fractions.

#### 3.2.1. G^−^ and G^+^ Peaks Profile Analysis

To quantify the separation efficiency, the full width at half maximum (FWHM) of the G^−^ peaks was measured, as summarized in Table 1. Metallic-enriched fractions (M1, M2, and M3) exhibited broader G^−^ peaks, with full width at half maximum (FWHM) values between 47.45 and 73.17 cm^−1^, reflecting a heterogeneous electronic composition. The broadening and asymmetry observed in the G^−^ peaks for metallic SWNTs are characteristic of the Breit–Wigner–Fano (BWF) band, which arises from electron–phonon coupling and the interaction between discrete phonon states and electronic continuum [32,33,34]. This behavior is unique to metallic SWNTs and further highlights their distinct electronic properties compared to semiconducting SWNTs. In contrast, the semiconducting-enriched fractions (S1, S2, S3) displayed narrower G^−^ peaks, with FWHM values ranging from 18.75 to 24.21 cm^−1^, which is consistent with a well-defined electronic structure. Fractions eluted with 5 wt% SDS (S1, S2, S3) showed FWHM values that were approximately half those of fractions eluted with 0.5 wt% SDS (M1, M2, M3). The reduction in FWHM across sequential samples highlights the progressive isolation of semiconducting SWNTs. The difference in peak positions between metallic and semiconducting SWNTs further corroborates the successful separation. Generally, metallic SWNTs with identical diameters exhibit G^−^ peaks at lower positions than semiconducting SWNTs [29]. However, as shown in Table 1, metallic samples M1 and M3 had higher G^−^ peak positions than some semiconducting samples, likely due to the presence of SWNTs with varying diameters, as suggested by the RBM peaks in Figure 4. Previous studies indicate that smaller-diameter SWNTs, which correspond to RBM peaks at higher wavenumbers, tend to display G^−^ peaks at lower wavenumbers [29,30]. The samples separated with 5 wt% SDS in Figure 4 exhibited RBM peaks at larger wavenumbers than those separated with 0.5 wt% SDS, suggesting that the G^−^ peaks in S1, S2, and S3 are influenced by SWNT diameter. Additionally, the intensity of G^−^ for each sample was divided by the intensity of G^+^ to represent the relative ratio of the two peak intensities. M1, M2, and M3 showed values ranging between 0.43 and 0.62, while S1, S2, and S3 exhibited relatively lower values, ranging between 0.20 and 0.30.

**Figure 7:**
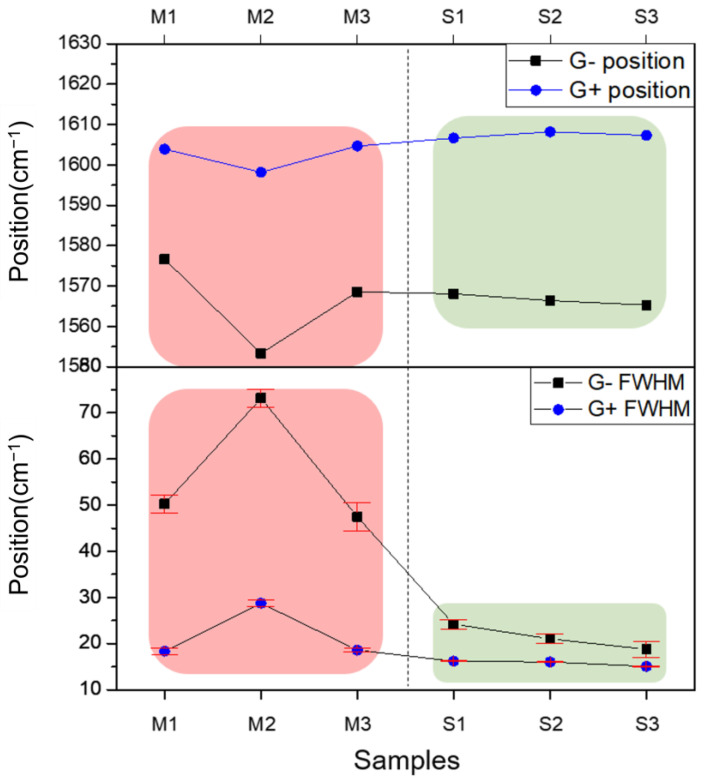
The position and FWHM of G^−^ and G^+^ peaks are compared across separated SWNT samples (M1–M3 for metallic-rich fractions and S1–S3 for semiconducting-rich fractions). The upper panel illustrates the G^−^ and G^+^ peak positions, highlighting systematic shifts due to electronic differences. The lower panel presents the FWHM for G^−^ and G^+^ peaks, showing broader G^−^ peaks for metallic-rich fractions.

The trends of G^−^ and G^+^ in the metallic and semiconducting samples are summarized in Figure 7, offering a comprehensive overview of the separation efficiency achieved via microfluidic gel chromatography combined with in situ Raman spectroscopy, as described in this study. The degree of separation is evident in the distinct differences in peak position, FWHM, and the relative intensity ratio (I_G_^−^/I_G_^+^), all of which clearly distinguish the electronic and structural characteristics of the two SWNT types. In particular, the pronounced broadening in the FWHM of G^−^ and the marked variations in I_G_^−^/I_G_^+^ ratios confirm the effectiveness of the microchannel-enabled separation technique. In the upper panel, the G^−^ peak positions for metallic-enriched fractions (M1, M2, and M3) are systematically lower than those for semiconducting-enriched fractions (S1, S2, and S3). This trend arises from the unique electronic density of states near the Fermi level in metallic SWNTs, underscoring the fundamental impact of electronic type on Raman spectral features. In contrast, the G^+^ peak remains relatively stable across all fractions, demonstrating its independence from electronic properties. The middle panel emphasizes the striking contrast in the FWHM of G^−^ peaks between the two types of SWNTs. The broader and more asymmetric G^−^ peaks in the metallic fractions, with FWHM values ranging from 47.45 to 73.17 cm^−1^, reflect the influence of the BWF line shape caused by electron–phonon coupling. By comparison, the semiconducting fractions exhibit narrower G^−^ peaks (18.75–24.21 cm^−1^), indicative of their uniform electronic structure and the progressive isolation achieved during separation. These observations collectively validate the microfluidic gel chromatography-enabled in situ Raman spectroscopy approach for selective electronic-type separation and analysis of SWNTs. Not only does this methodology effectively isolate metallic and semiconducting SWNTs, but it also reveals subtle vibrational and electronic interactions inherent to each type. The pronounced differences in G peak characteristics—spanning the position, width, and intensity ratio—underscore the critical role of electron–phonon interactions and diameter variation in defining the Raman spectral behavior of SWNTs.

#### 3.2.2. G^−^/G^+^ Intensity Ratio Analysis for Elution Dynamics in SWNT Separation

**Figure 8:**
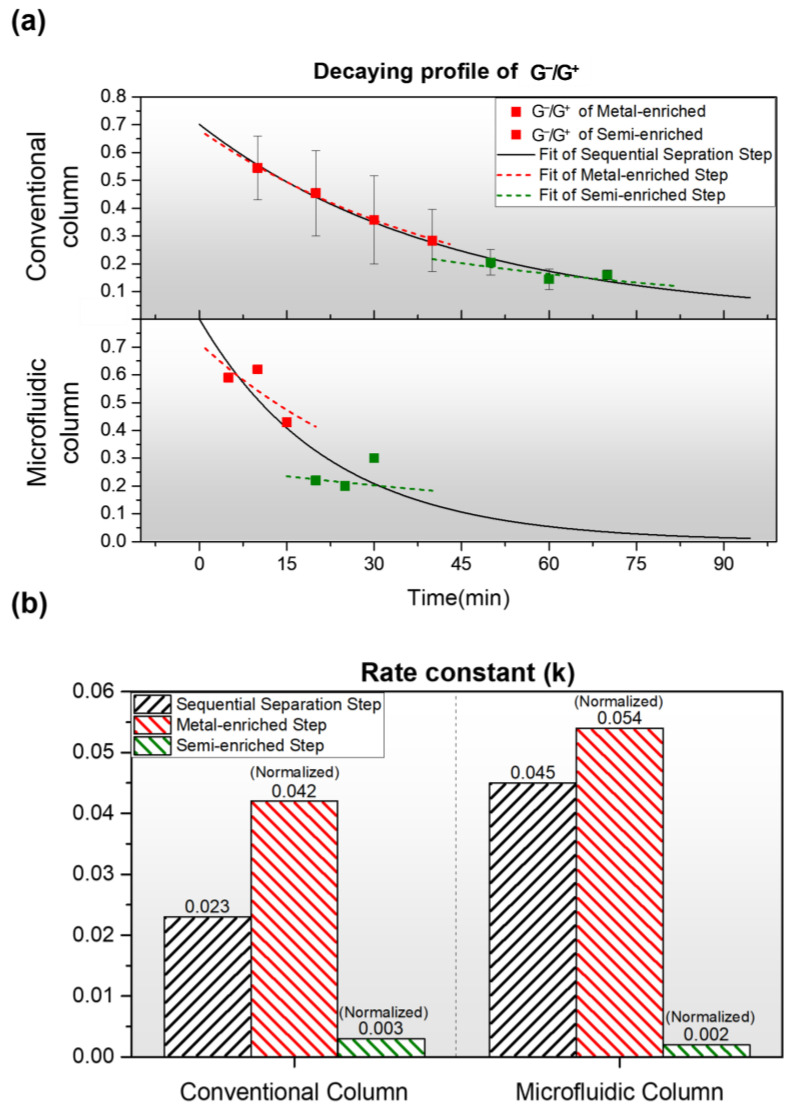
Analysis of SWNT elution dynamics based on the G^−^/G^+^ intensity ratio, comparing conventional and microfluidic columns. (**a**) Decay profiles of the G^−^/G^+^ ratio over sequential separation time, illustrating the differences between metal-enriched and semi-enriched fractions across the two separation methods. The data were fitted to an exponential decay model to derive the rate constant (k) for each fraction. (**b**) Bar graphs showing the rate constants ($k_{normalized}$) for each step and column type, normalized by SDS concentration, demonstrate a faster elution rate for the metallic-rich fractions.

Figure 8 provides an integrated view of the elution dynamics and separation characteristics of metallic- and semiconducting-enriched SWNT fractions. The analysis is based on the G^−^/G^+^ intensity ratio measured via Raman spectroscopy, comparing data between the conventional column and the microfluidic column of this study. For both conventional and microfluidic columns in Figure 8a, the overall sequential separation step showed a decaying curve over time. Metallic-rich fractions exhibit a steeper decay curve, indicative of faster elution rates. In contrast, the semiconducting-rich fractions show a more gradual decay, consistent with their stronger interaction with the gel matrix due to electronic differences. In the microfluidic column, metallic-rich samples display significantly higher ratios (0.43–0.62), whereas semiconducting-rich fractions show much lower ratios (0.20–0.30). Also in the conventional column, metallic- and semiconducting-enrich samples display higher ratios (0.30–0.54) and lower ratios (0.14–0.20), respectively. These differences between the metallic- and semiconducting-rich fractions of both columns are consistent with the dominance of the broadened G^−^ peak, reflecting their distinct Raman characteristics. These values were analyzed over time to calculate rate constants for elution in order to assess SWNT purity while accounting for the differing SDS concentrations used during separation.

### 3.3. Methodology for Rate Constant Calculation

To derive the rate constants (k), the decay in the G^−^/G^+^ intensity ratio over time was modeled using a single exponential decay function, under the assumption that the elution dynamics follow a first-order process. The measured data were fit to Equation (1):

(1) $$\frac{I_{G^{-}}}{I_{G^{+}}}\left(t\right)=\frac{I_{G^{-}}}{I_{G^{+}}}\left(0\right)\cdot e^{-kt}$$

where, $\frac{I_{G^{-}}}{I_{G^{+}}}\left(t\right)$ is the G^−^/G^+^ intensity ratio at time *t*, $\frac{I_{G^{-}}}{I_{G^{+}}}\left(0\right)$ is the initial ratio, and *k* is the rate constant of elution. For both the conventional and microfluidic columns, the rate constants for the overall separation steps over time were calculated. The rate constant (k) for the overall steps of the microfluidic column was $0.045\;{min}^{-1}$, which is higher than the $0.023\;{min}^{-1}$ of the conventional column. This difference is attributed to the faster elution speed or efficiency of the microfluidic column, enabled by its higher diffusion rate and specific volume at the microscale.

Additionally, $k_{metal}$ and $k_{semi}$ were derived from the metallic- and semiconducting-rich fractions for each column. In the conventional column, the decay dynamics yielded $k_{metal}=0.021\;{min}^{-1}$ and $k_{semi}=0.015\;{min}^{-1}$, while the microfluidic column yielded $k_{metal}=0.027\;{min}^{-1}$ and $k_{semi}=0.010\;{min}^{-1}$. Both types of columns displayed higher rate constants for the metallic fractions reflecting their rapid elution due to weaker interactions with the gel matrix compared to the semiconducting fractions.

### 3.4. Normalization of Rate Constants

To emphasize the intrinsic elution dynamics independent of SDS concentration, the rate constants were normalized, as shown in Figure 8b. To account for the 10-fold difference in SDS concentration between the metallic- and semiconducting-enriched fractions (0.5 wt% vs. 5 wt%), the rate constants were normalized using Equation (2):

(2) $$k_{normalized}=\frac{k}{\left[SDS\right]}$$

where [SDS] represents the SDS concentration in wt%. The normalized rate constants for the conventional column were $k_{normalized,metal}=0.0{42\;min}^{-1}\cdot {wt}^{-1}\%$ and $k_{normalized,semi}=0.003\;{min}^{-1}\cdot {wt}^{-1}\%$. In the microfluidic column, they were $k_{normalized,metal}=0.054\;{min}^{-1}\cdot {wt}^{-1}\%$ and $k_{normalized,semi}=0.002\;{min}^{-1}\cdot {wt}^{-1}\%$. Both columns exhibited distinct normalized rate constants for different electronic types, with the microfluidic column showing larger differences compared to the conventional column. The clear separation of normalized rate constants between metallic- and semiconducting-enriched fractions correlates with their respective purities. The purity of each fraction is closely tied to the elution dynamics, as faster elution typically corresponds to lower gel–matrix interaction and higher metallicity. Conversely, the slower elution of semiconducting SWNTs aligns with their greater affinity for the gel and higher electronic uniformity. These findings validate the use of G^−^/G^+^ ratio decay as a reliable metric for quantifying SWNT separation efficiency and purity. The G^−^/G^+^ ratio decay trend observed across the entire range consistently serves as a reliable indicator for quantifying SWNT separation efficiency and purity, regardless of the column type.

Furthermore, the unique microfluidic column developed in this study demonstrated a rate constant difference that was twice as large as that of the conventional column throughout the process, significantly amplifying the relative disparity in rate constants between metallic and semiconducting SWNTs. This analysis validates the effectiveness of the microfluidic gel chromatography approach in achieving continuous electronic-type separation of SWNTs and underscores the importance of considering both experimental parameters (e.g., SDS concentration) and the intrinsic properties of SWNTs when interpreting rate constants and purity metrics.

## 4. Conclusions

This study has presented and validated an innovative PDMS-based microfluidic gel chromatography system designed to address the challenges of precise SWNT separation at the microscale. By exploiting the selective interactions between SWNTs, dispersants, and the gel matrix, this platform achieved the efficient and sequential isolation of metallic and semiconducting SWNTs with remarkable accuracy. Validation through G^−^/G^+^ ratios and G-band spectral shifts provided critical insights into the elution dynamics unique to each SWNT type. Moreover, an SDS concentration-dependent analysis revealed distinct trends in rate constants, highlighting faster elution kinetics for metallic SWNT fractions compared to their semiconducting counterparts and further establishing the platform’s ability to effectively differentiate SWNTs by their electronic properties. To the best of our knowledge, this study is the first to introduce microfluidic gel chromatography for SWNT separation. While previous research has extensively utilized conventional gel chromatography for nanomaterial separation, microfluidic systems have not been employed in this context until now. Our work bridges this gap by pioneering the integration of microfluidic gel chromatography with real-time in situ Raman spectroscopy, enabling simultaneous separation and electronic characterization with unparalleled efficiency. This approach offers new perspectives on the interplay between SWNT electronic properties and gel-based retention behaviors, setting a foundation for future advancements in nanomaterial purification technologies.

The scalability and adaptability of this PDMS-based microfluidic platform make it a robust and versatile tool for both fundamental research and industrial applications. Its compatibility with spectroscopic monitoring and its potential for high-throughput implementation provide a framework for addressing challenges in nanomaterial purification. By bridging the gap between microscale precision and large-scale practicality, this methodology advances SWNT separation and paves the way for future innovations in gel chromatography and microfluidic systems. The continued refinement of this platform promises to unlock new capabilities in chirality-specific separations and analytical techniques, fostering transformative progress in the field of nanotechnology.

## 5. Patents

Method for separating single-wall carbon nanotubes [KR Patent Application No. 10-2024-0077633].

## Figures and Tables

**Table 1:** Peak positions and full width at half-maximum (FWHM) values for the G^−^ and G^+^ peaks of metallic-rich (M1, M2, and M3) and semiconducting-rich (S1, S2, and S3) SWNT fractions. The intensity ratio between G^−^ and G^+^ peaks (G^−^/G^+^) is also included for each sample, providing insights into the structural and electronic type-specific characteristics of the separated SWNTs. Each sample designation represents the sequential elution step during the separation process.

Separated Fractions	G^−^ Positon (cm^−1^)	G^+^ Position (cm^−1^)	G^−^ FWHM (cm^−1^)	G+ FWHM (cm^−1^)	Intensity Ratio of G^−^/G^+^
M1	1576.65	1603.89	50.24	18.35	0.59
M2	1553.33	1598.18	73.17	28.74	0.62
M3	1568.54	1604.67	47.45	18.57	0.43
S1	1568.05	1606.63	24.21	16.17	0.22
S2	1566.39	1608.18	21.05	16.00	0.20
S3	1565.30	1607.31	18.75	15.04	0.30

## Data Availability

The original contributions presented in the study are included in the article; further inquiries can be directed to the corresponding author.

## Supplementary Materials

The following supporting information can be downloaded at https://www.mdpi.com/article/10.3390/polym17010093/s1, This includes detailed abbreviations utilized throughout the manuscript (defined in Table S1) and comprehensive spectroscopy data obtained from separated SWNT fractions. In Figure S1, UV-vis spectroscopy results for metallic and semiconducting SWNTs are displayed in (a) and (b), while RBM peaks from Raman spectroscopy are depicted in (c) and (d). Additionally, (e) and (f) highlight the G^−^ and G^+^ peaks of Raman spectroscopy, and (g) presents photoluminescence excitation (PLE) spectroscopy data for semiconducting SWNTs.
