# Supplementary material for: In Situ Raman Spectroscopy-Enabled Microfluidic Gel Chromatography for Revealing Real-Time Separation Dynamics of Single-Walled Carbon Nanotubes
Source: Polymers (Basel). 2025 Jan 1;17(1):93. doi: 10.3390/polym17010093 (PMC11723415; doi:10.3390/polym17010093)
Supplement: Supplementary file 1 [file polymers-17-00093-s001.zip › polymers-3377175-supplementary.pdf]

Supplementary Materials

In-Situ Raman Spectroscopy-Enabled Microfluidic Gel Chro-matography for Revealing Real-Time Separation Dynamics of Single-Walled Carbon Nanotubes

Byeongji Beom[1,†], Seung-Chan Jung[1,†], Wonjun Jang [1,†], , Jong-Keon Won [1], Jihoon Jeong [1], Yu-Jeong Choi [1], Man-Ki Moon [1], and Jae-Hee Han [1,*]

[1] Department of Materials Science and Engineering, Gachon University, Seongnam 13120, Republic of Korea

* Correspondence: jhhan388@gachon.ac.kr (J.-H.H.); Tel.: (+82-31-750-8689)

† These authors contributed equally to this work.

Table S1. Abbreviation of terms which used in both the main text and Supplementary Materials.

Abbreviation	Meaning
M1	Metallic-enriched fractions 1
M2	Metallic-enriched fractions 2
M3	Metallic-enriched fractions 3
S1	Semiconducting-enriched fractions 1
S2	Semiconducting-enriched fractions 2
S3	Semiconducting-enriched fractions 3
G^- peak	G-peak observed around 1560 cm^{-1}
G^+ peak	G-peak observed around 1590 cm^{-1}
HiPco	High Pressure Carbon Monoxide (Synthesis method)
RBM	Radial Breathing Mode
FWHM	Full Width at Half Maximum
I_{G^-}/I_{G^+}	Intensity ratio of G^-/G^+ band
K_{metal}	Rate constant of metal-enriched fraction
K_{semi}	Rate constant of semiconducting-enriched fraction
$K_{normalized}$	Normalized Rate constant
$K_{normalized,metal}$	Normalized Rate constant of metal-enriched fraction
$K_{normalized,semi}$	Normalized Rate constant of semiconducting-enriched fraction

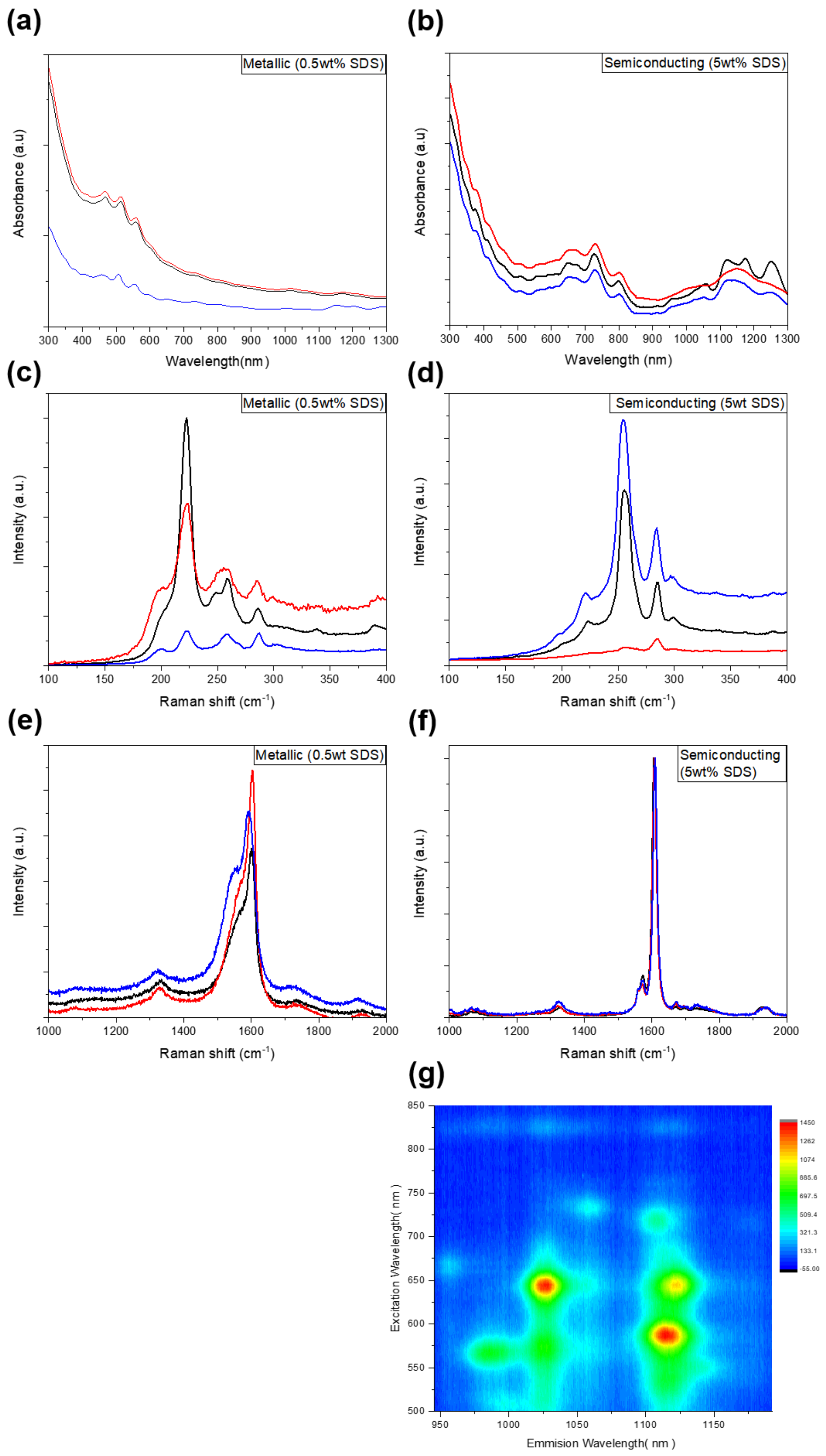

Figure S1. Spectrocopy data of separated SWNTs by gel chromaotgraphy in the conventional column. UV-vis spectroscopy data are shown in (a) metallic and (b) semiconducting SWNTs. The RBM peaks of Raman spectroscopy are shown in (c) metallic and (d) semiconducting SWNTs. Both D and G peaks of Raman spectroscopy are shown in (e) metallic and (f) semiconducting SWNTs. The data for photoluminescence exciation (PLE) spectroscopy of semiconducting SWNTs is shown in (g).
